# Relationships between Alcohol Use, Musculoskeletal Pain, and Work-Related Burnout

**DOI:** 10.3390/medicina58081022

**Published:** 2022-07-29

**Authors:** Yong-Hsin Chen, Chih-Jung Yeh, Lung-Fa Pan, Gwo-Ping Jong

**Affiliations:** 1 Department of Public Health, Chung Shan Medical University, Taichung 402, Taiwan; a6328539@gmail.com (Y.-H.C.); alexyeh@csmu.edu.tw (C.-J.Y.); 2 Department of Occupational Safety and Health, Chung Shan Medical University Hospital, Taichung 402, Taiwan; 3 Department of Medical Imaging and Radiological Science, Central Taiwan University of Science and Technology, Takun, Taichung 406, Taiwan; panlungfa@gmail.com; 4 Department of Cardiology, Taichung Armed Forces General Hospital, Taichung 411, Taiwan; 5 Department of Internal Medicine, Chung Shan Medical University Hospital, Chung Shan Medical University, Taichung 402, Taiwan

**Keywords:** alcohol use, musculoskeletal pain, work-related stress, burnout

## Abstract

*Background and Objectives*: Burnout affects approximately half of all nurses, physicians, and other clinicians. Alcohol use may impair performance in work-related tasks, leading to decreased productivity and morale. The present study’s aim was to determine whether a causal relationship existed between alcohol use, work-related burnout (WB), and musculoskeletal pain. *Materials and Methods*: A total of 1633 members from a hospital affiliated with a medical university in Taichung, Taiwan, completed questionnaires in 2021, where 1615 questionnaires were declared valid. Questionnaires were used to obtain information on basic demographic variables, and the Nordic Musculoskeletal Questionnaire and Copenhagen Burnout Inventory were used. Statistical analyses were performed using SAS Enterprise Guide 6.1 software, and significance was set at *p* < 0.05. *Results*: Work experience, being married, parenthood, leisure activities with family and friends, and regular weekly exercise were negatively associated with WB. In addition, overtime work, irregular and regular shift work, the physician and nurse medical profession, chronic disease (heart disease, diabetes, etc.), neck and both shoulders pain (NBSP), both ankles pain (BAP), and alcohol use frequency (AUF) were positively associated with WB. NBSP could explain the residual effect of AUF on WB. AUF was determined to mediate the relationship between NBSP and WB. In addition, NBSP was found to mediate the relationship between AUF and WB. *Conclusions*: The individuals who used alcohol to cope with NBSP or those with NBSP who often consumed alcohol had worsened WB due to a vicious circle of musculoskeletal pain and alcohol use. Therefore, medical staff should not consider alcohol use as an option to reduce burnout.

## 1. Introduction

Burnout was first described in 1974 by the clinical psychologist Herbert Freudenberger, who borrowed the term from drug-addict slang [1]. Burnout refers to physical, emotional, and mental exhaustion resulting from long-term work situations [2]. To measure burnout, Christina Maslach proposed the Maslach Burnout Inventory in 1981, and the extent of an individual’s symptoms in each dimension is measured on the basis of the three subscales of emotional exhaustion, depersonalization, and a diminished sense of personal accomplishment [3]. The Copenhagen Burnout Inventory (CBI) developed by researchers from Denmark is another scale used to measure burnout [4]. The CBI considers exhaustion as the core of the burnout concept and includes three scales, namely, the personal burnout scale, work-related burnout (WB) scale, and client burnout scale, which can be separately used to measure burnout in different settings (not only for service professions); the questionnaire is developed in such a manner that it is suitable for individuals from all occupations [4]. In contrast with personal and client burnout, WB is defined in the CBI as the degree of physical and psychological fatigue and exhaustion that is perceived by the person as related to paid work of some kind [4].

The severe clinical presentations of burnout include emotional exhaustion, physical fatigue, cognitive impairments, disturbed sleep, and functional impairment [5,6]. A combination of stressors due to long-term work and nonwork on individuals often contribute to clinical burnout [7], which could lead to sleep disturbances, depression, or anxiety disorders [5]. Burnout affects approximately half of all nurses, physicians, and other clinicians [8]. Among them, one-tenth of the nurses worldwide suffer high burnout symptoms [9] and 48.7% of German clinicians meet the criteria for burnout [10]. Notably, clinical burnout is related to a reduced ability to work [7]. Burnout is responsible for high physician turnover and reduced clinical hours, which cause total losses of approximately USD 4.6 billion each year [11]. Notably, burnout also affects the patient-related quality of care [10].

In the United States, 13% of the total workforce loss is associated with body pain conditions and costs an estimated USD 61.2 billion per year [12]. A large study conducted in the Netherlands demonstrated that the top three self-reported musculoskeletal pain sites were the lower back, shoulder, and neck [13]. Moreover, studies reported diverse risk factors for musculoskeletal pain, including long work hours [14], occupational stress [15], alcohol consumption [16], sleep duration [17], and chronic diseases (CDs) [18].

Alcohol use contributes to around 4% of the global burden of disease [19] and is related to premature death, where the major causes are injury, alcoholic liver disease, heart disease and stroke, cancers, and gastrointestinal disease [20]. Alcohol use may impair performance in work-related tasks, leading to decreased productivity and morale [21] and an increased occupational injury risk [22]. Many individuals consume alcohol to alleviate stress caused by working overtime [23] and even to cope with pain [24].

Individuals commonly tend to adopt an alcohol consumption strategy to cope with pain, psychological, or physical troubles. Based on this, the present study asked questions about alcohol use, MS pain, and burnout to establish (1) whether a causal relationship exists between alcohol use, WB, and musculoskeletal pain, and (2) the role of alcohol use in the relationship between musculoskeletal pain and WB?

## 2. Methods

### 2.1. Study Population

In this observational and cross-sectional study, we included 2531 employees from a hospital affiliated with a medical university in Taichung, Taiwan, in 2021. The study protocol was approved by the institutional review board of Chung Shan Medical University Hospital on 25 August 2021 (no. CS1-21108).

### 2.2. Study Measures

The QR-code-linked questionnaires were sent to all eligible participants by email. Among the 2531 members, 1633 (64.52%) completed questionnaires. After exclusion due to missing data, 1615 (63.81%) questionnaires were determined to be valid. The questionnaires were used to obtain information on basic demographic variables, family factors, living habits, work-related factors, and physical health factors. In addition, we used the Nordic Musculoskeletal Questionnaire (NMQ) and the WB scale.

Regarding the education level, the response options were “below high school”, “Bachelor”, “Master”, and “PhD”. The response options for marriage status were “married” and “others”. In terms of family factors, we examined whether the respondents were parents by using the following response options: “without child”, “one child”, “two children”, “three children”, and “over three children”. Raising at least one child was reclassified as a new variable called “parenthood”.

Relationships with family and friends were also evaluated in the questionnaire. For the question “Whether participants engage in leisure activities with family or friends in vacation time?”, the Likert scale method was used, where the response options were distinguished as “always”, “often”, “sometimes”, “seldom”, and” never”; these were scored as 100, 75, 50, 25, and 0 points, respectively. The data was an ordinal scale that could be suitable for parametric tests (such as *t*-tests, analysis of variance, Pearson correlations, and regression), even when statistical assumptions were violated (such as normal distribution of data) [25]. An item related to the presence of a listed CD was included in the questionnaire, and the presence of one or more diseases was classified as a “yes” response. Regarding AU in the past month, the response options were “always”, “often”, “sometimes”, “seldom”, and “never”; these responses were scored as 100, 75, 50, 25, and 0 points, respectively. Finally, the mean value was considered as the new variable AU frequency (AUF). The response options for the sleep duration (SLD) were classified as <5, 5–6, 6–7, 7–8, or >8 h per day; these were reclassified as SLD < 6 h and SLD > 6 h per day. The response options for exercise habit were “at least once a day”, “at least once a week”, “at least once a month”, “less than once a month”, or “never”. Exercising at least once a day or week was reclassified as regular exercise weekly (REW). The response options for the question on overtime (OT) were “seldom”, “less than 45 h per month”, “45–80 h per month”, and “more than 80 h per month”. The responses were reclassified as seldom OT and experiencing OT (including less than 45 h, 45–80 h, and more than 80 h per month). The possible responses to the question on the shift schedule were “day shift work”, “night shift work”, “irregular shift (IRS) work”, and “regular shift (RS) work”.

We adopted the NMQ that was modified and translated by the Taiwan Institute of Occupational Safety and Health, which includes questions on the presence of pain attributable to work-related factors in the preceding year and at pain sites. The options for pain sites were the neck, left shoulder, right shoulder, upper back, waist or lower back, left elbow, right elbow, left wrist, right wrist, left hip/thigh/buttock, right hip/thigh/buttock, left knee, right knee, left ankle, and right ankle. If a participant answered “yes” to a question on the experience of work-related pain in the preceding year, they were required to indicate its occurrence frequency: every day, once a week, once a month, once every half year, or at least once every half year (100, 80, 60, 40, and 20 points, respectively).

We used the Chinese version of the CBI [26], which was reported to be a reliable and valid tool (the Cronbach’s alpha value was over 0.84 for males and females) for the assessment of burnout and measuring WB. The seven items for measuring WB were as follows:

1. “Is your work emotionally exhausting?”
2. “Do you feel burnt out because of your work?”
3. “Does your work frustrate you?”
4. “Do you feel worn out at the end of the working day?”
5. “Are you exhausted in the morning at the thought of another day at work?”
6. “Do you feel that every working hour is tiring for you?”
7. “Do you have adequate energy for family and friends during leisure time?”

The response options were “always”, “often”, “sometimes”, “seldom”, and “never or almost never”, and these were scored as 100, 75, 50, 25, and 0, respectively, except for item 7, which was inversely scored (i.e., the responses were scored as 0, 25, 50, 75, and 100, respectively); the calculated mean value indicated the WB level for the participants.

### 2.3. Data Analysis

Factor analysis [27] was conducted using the NMQ results to determine the underlying variables that explained most of the questionnaire. A univariate linear regression model was used to examine the associations between the dependent variables (DVs) and independent variables (IVs). Multiple linear regression was conducted to determine whether the adjustment for variables significantly affected the associations between IVs and DVs. Mediation effects were analyzed using the strategy proposed by Baron and Kenny [28], in which (1) the IV significantly affects the mediator (first-stage effect), (2) the IV significantly affects the DV in the absence of the mediator, (3) the mediator exerts a significant unique effect on the DV (second-stage effect), and (4) the effect of the IV on the DV weakens upon the addition of a mediator to the model. Among them, item (2) is only recommended but not required [29]. The formulas are as follows:

$$Y=b_{01}+cX$$

$$M=b_{02}+aX$$

$$Y=b_{03}+c^{\prime }X+bM$$

where *X* is an IV, Y is a DV, M is the adjusted variable (i.e., the mediating factor), *a* is the linear regression coefficient of *X* against M, *b* is the linear regression coefficient of M against Y, *c* is the linear regression coefficient of *X* against Y, and *c*′ is the linear regression coefficient of *X* against Y with M as the adjusting variable. The standard errors of *a* and *b* are represented by *s_a_* and *s_b_*, respectively. The formula for the Sobel test is as follows:

$$Z=\frac{a\times b}{\sqrt{b^{2}s_{a}{}^{2}+a^{2}s_{b}{}^{2}}}$$

The results exceeding |1.96|, |2.57|, and |3.90| (for a two-tailed test) are significant at α = 0.05, 0.01, and 0.0001, respectively.

The mediation proportion is defined as the dimensionless proportion of the effect of an IV on a DV mediated through the mediation factor, whose formula is as follows [30]:

$$MP=\frac{a\times b}{c^{\prime }+a\times b}$$

Analyses were performed using SAS Enterprise Guide 6.1 software (SAS Institute Inc., Cary, NC, USA), and significance was set at *p* < 0.05.

## 3. Results

### 3.1. Musculoskeletal Pain Sites and Factor Analysis of the Nordic Musculoskeletal Questionnaire

The prevalences of musculoskeletal pain in a year in both shoulders, the neck, the waist, the lower back, and the upper back were 43.09%, 36.22%, 27.93%, and 16.90%, respectively (Table 1). The mean frequency scores of neck, waist or lower back, right shoulder, left shoulder, and upper back pain were 26.76 ± 37.64, 20.20 ± 34.72, 17.64 ± 33.89, 15.07 ± 31.62, and 12.90 ± 29.77, respectively. According to the principle proposed by Hair and Anderson (1995) [27], factors 1 and 2 were retained because their vector values exceeded 1. Although the eigenvalue of factor 3 was lower than 1, factor 3 was retained to ensure the maximum ability to explain the questionnaire. The factor loadings were converted into standardized scoring coefficients through varimax rotation. The relatively large factor loading values for factors 1, 2, and 3 corresponded to frequency scores for the neck and both shoulder pain (NBSP), both ankle pain (BAP), and both knee pain (BKP), respectively.

### 3.2. Statistical Results of Response Options for Every Work-Related Burnout Item

Table 2 presents the statistical results of the response options for every WB item. The Cronbach’s alpha value of the WB scale was 0.87. The mean values of all items for measuring WB are shown below. The mean values and standard deviations of all items for the WB scale are shown below.

As presented in Table 3, work experience was negatively associated with WB (β = −0.23, *p* < 0.0001) and AUF (β = −0.21, *p* < 0.01). Married status and parenthood were protective factors against WB (β = −4.30 and −4.86, respectively; *p* < 0.0001 for both) and AUF (β = −2.68 and −3.10, respectively; *p* < 0.05 for both). Engaging in leisure activities with family and friends was negatively associated with WB (β = −0.14, *p* < 0.0001) but not with AUF (β = −0.00, *p* > 0.05). Regular weekly exercise was a protective factor against WB (β = −5.60, *p* < 0.0001); however, it was not significantly associated with AUF (β = 1.72, *p* > 0.05). SLD < 6 h per day was significantly positively associated with WB (β = 6.27, *p* < 0.0001) and AUF (β = 3.11, *p* < 0.05). The participants with a master’s degree or above reported a lower level of WB (β = −2.21, *p* < 0.05) than those with a university or below university degree; however, having a master’s degree or above was not significantly associated with AUF (β = 2.17, *p* > 0.05). OT work was a risk factor for WB (β = 8.88, *p* < 0.0001) and AUF (β = 4.33, *p* < 0.001). A significant difference in WB was noted between different shift schedules. IRS and RS work significantly increased WB (β = 8.87 and 6.23, respectively; *p* < 0.0001 for both). Regarding professional fields, physicians (β = 9.89, *p* < 0.0001) and nurses (β = 8.44, *p* < 0.0001) reported higher levels of WB. Moreover, the AUF was significantly higher in the physicians (β = 9.96, *p* < 0.0001). The presence of a CD was positively associated with WB (β = 3.47, *p* < 0.0001) and AUF (β = 3.01, *p* < 0.05). Regarding musculoskeletal pain, NBSP was closely associated with WB (β = 6.30, *p* < 0.0001) and AUF (β = 2.48, *p* < 0.01). In addition, BAP was associated with WB (β = 1.44, *p* < 0.01).

### 3.3. The Association between Alcohol Use, Musculoskeletal Pain, and Work-Related Burnout

As presented for M_0_ in Table 4, AUF was closely associated with WB (β = 0.07, *p* < 0.0001). After an adjustment for work experience, marriage status, parenthood, leisure activity with family and friends, exercise habit weekly, SLD, education degree, OT work per month, shift schedules, professional fields, and CD in the M_1_ model, we observed that AUF was still associated with WB (β = 0.04, *p* < 0.01). In the M_2_ model, the residual effect of AUF on WB could be fully explained by NBSP (β = 0.03, *p* > 0.05). Mediation analysis was performed (Table 5) to determine whether a causal relationship existed between WB, AUF, and NBSP.

As presented in Table 5, AUF was determined to be a mediator (Z = 2.07, *p* < 0.05) of the relationship between NBSP and WB, with the mediation proportion being only 1.95%. In addition, NBSP was determined to be a mediator (Z = 3.25, *p* < 0.01) of the relationship between AUF and WB, with the mediation proportion being 27.08%.

## 4. Discussion

Our study’s results suggested that alcohol use was associated with increased NBSP, and both were closely related to increased WB. According to the mediation models, alcohol use mediated the relationship between NBSP and WB, causing worse WB. In addition, NBSP was a mediation factor of alcohol use, causing worse WB. Based on this, in response to the two research aims posed in the Introduction: (1) a causal relationship existed between alcohol use, WB, and musculoskeletal pain, and (2) alcohol use was a mediation factor between musculoskeletal pain and WB that increased WB.

Previous studies reported various causes of burnout. For example, a systematic review of the literature on burnout revealed that inexperienced psychotherapists easily experienced burnout due to a sense of hopelessness and an inability to reach idealistic expectations and standards [31]. In this study, work experience was found to be a protective factor against WB (β = −0.23, *p* < 0.0001; Table 3).

Family members and friends play a vital role in preventing burnout [32]. For instance, health workers reported that they could minimize burnout by obtaining support from family [33]. As demonstrated in Table 3, being married (β = −4.30, *p* < 0.0001) and parenthood (β = −4.86, *p* < 0.0001) were negatively associated with WB. These results indicated that family members could effectively reduce the level of WB; this finding is consistent with those of previous studies. Participating in leisure activities can relieve stress, help cope with emotional stress, and moderately maintain physical and mental health [34]. The same finding was observed in this study: engaging in leisure activities with family and friends was negatively associated with WB (β = −0.14, *p* < 0.0001).

Physiological changes resulting from physical activity can reduce individuals’ sensitivity to chronic stress [35]. Thus, engaging in physical activity can lead to faster recovery after experiencing a stressful situation, and thus, reduce the risk of burnout [36]. Our results revealed that regular weekly exercise effectively reduced WB (β = −5.60, *p* < 0.0001).

Burnout development was closely related to considerably less sleep (<6 h) [37] and disturbed sleep [38]. Similar to the findings of previous studies, our results revealed that the participants with SLD < 6 h/day reported a higher level of WB than those with SLD > 6 h/day (β = 6.27, *p* < 0.0001).

OT work hours are closely correlated with burnout development in a dose-dependent manner [39]. IRS work is related to a significantly higher level of burnout [40]. The same phenomena were observed in our study: OT (β = 8.88, *p* < 0.0001) and IRS work (β = 8.87, *p* < 0.0001) were significantly associated with WB.

Burnout was observed to be markedly higher among practicing physicians than individuals in other careers after adjustment for work hours and other factors [41]. The present study indicated that the physicians reported a higher level of WB than those employed in other fields (Table 3). This result is consistent with those of previous studies.

Burnout is an independent risk factor for coronary heart disease [42] and type 2 diabetes [43]. Similar findings are presented in Table 3. The participants who had at least one CD reported a higher level of WB than those without CD (β = 3.47, *p* < 0.0001).

The onset of regional neck/shoulder and/or low back pain was associated with an increased risk of burnout [44]. In the present study, NBSP was significantly related to WB (β = 6.30, *p* < 0.0001). Overall, the increased occurrence frequency of neck and shoulder pain increased WB.

Burnout was strongly associated with alcohol abuse or dependence among American surgeons [45] and was significantly positively associated with higher AU among doctors, nurses, and residents [46]. The present study demonstrated that AUF was significantly associated with work-related burnout in a univariate linear regression (Table 3; M_0_: β = 0.07, *p* < 0.0001). Even after adjusting for other risk factors, we observed that this association was still significant (Table 3; M_1_: β = 0.04, *p* < 0.01). However, this association did not become significant after the addition of an extra adjusted variable, namely, NBSP, in the M_1_ model (M_2_ model). NBSP fully explained the residual effect of AUF on WB after an adjustment for other risk factors.

Previous studies demonstrated a close relationship between musculoskeletal pain, burnout, and alcohol use. Musculoskeletal pain is associated with the frequency of drinking, and the association between alcohol consumption and pain is curvilinear [47]. For example, moderate alcohol consumption was associated with a decreased risk of disabling chronic back or neck pain [48] and chronic widespread pain [49]. Excessive alcohol use may cause the development of chronic pain by increasing the risk of traumatic injury and deleterious effects on the musculoskeletal system [50]. To determine the causal relationship between AUF, NBSP, and WB, three variables were modeled and the results are presented in Table 5.

The mediation model (Table 5) demonstrated that AUF mediated the relationship between NBSP and WB and that NBSP mediated the relationship between AUF and WB. Some individuals who often reported NBSP used alcohol to cope with pain; however, this strategy worsened their WB due to increased alcohol use (the mediation proportion was only 1.95%). In addition, the individuals who often consumed alcohol often reported NBSP, which further increased WB due to worsening musculoskeletal pain (the mediation proportion reached 27.08%). These relationships formed a vicious circle of WB for medical staff who used alcohol and had musculoskeletal pain.

The present study only surveyed the frequency of alcohol use in the past month and ignored the amount of alcohol intake per day by the participants. This could mean that alcohol’s effects on burnout and musculoskeletal pain were weakened. The severity of musculoskeletal pain was also ignored since we could not further explore whether the dose–response relationship between alcohol use and musculoskeletal pain or burnout existed. Notably, our study was conducted during the COVID-19 pandemic. It was reported that over 50% of healthcare professionals reported burnout symptoms during the COVID-19 pandemic, which was mainly caused by contacting patients, supply shortages, and work impacting household activities [51]. However, our study’s variables did not include these factors.

## 5. Conclusions

Work experience, marriage, and parenthood were negatively associated with WB and the frequency of alcohol use. A sleep duration of less than 6 h per day, overtime work, physician medical profession, suffering from CDs, and increased NBSP were positively associated with WB and frequency of alcohol use. Leisure activity with family and friends, regular exercise weekly, and a master’s degree or above were negatively associated with WB. Shift work, nurse medical profession, and increased BAP were positively associated with WB.

Alcohol use and NBSP were closely associated, and both were independent risk factors for WB. Mediation models indicated that the individuals who used alcohol to cope with NBSP or those with NBSP who often consumed alcohol had worsened WB due to a vicious circle of musculoskeletal pain and alcohol use. Therefore, medical institutions should positively encourage that staff quit drinking or drink in moderation. In particular, individuals who suffer from neck and shoulder pain should not consider alcohol use to cope with burnout symptoms.

## Figures and Tables

**Table 1:** Musculoskeletal pain sites and factor analysis of the Nordic musculoskeletal questionnaire.

MS Pain Site	MS Pain Subjects	Prevalence (%)	Frequency Score	Factor Loading		
			Mean ± SD	Factor 1	Factor 2	Factor 3
Neck	585	36.22	26.76 ± 37.64	***0.33***	−0.02	−0.03
Left shoulder	325	20.12	15.07 ± 31.62	***0.33***	−0.01	−0.01
Right shoulder	371	22.97	17.64 ± 33.89	***0.33***	0.02	−0.07
Upper back	273	16.90	12.90 ± 29.77	0.17	0.00	−0.01
Waist or lower back	451	27.93	20.20 ± 34.72	0.08	−0.04	0.03
Left elbow	70	4.33	3.29 ± 16.26	−0.05	−0.04	−0.05
Right elbow	113	7.00	5.33 ± 20.43	−0.04	−0.04	−0.02
Left wrist	77	4.77	3.72 ± 17.38	−0.05	0.00	0.01
Right wrist	162	10.03	7.51 ± 23.66	−0.03	−0.03	−0.02
Left hip/thigh/buttock	67	4.15	3.12 ± 15.64	−0.05	−0.07	−0.01
Right hip/thigh/buttock	68	4.21	3.17 ± 15.83	−0.02	−0.04	−0.06
Left knee	80	4.95	3.78 ± 16.98	−0.05	−0.07	***0.51***
Right knee	88	5.45	4.17 ± 18.05	−0.02	−0.04	***0.45***
Left ankle	29	1.80	1.26 ± 10.10	−0.02	***0.49***	−0.05
Right ankle	25	1.55	1.10 ± 9.58	−0.02	***0.54***	−0.05
		Eigenvalues		4.93	1.55	0.68
		Explained variation (%)		57.59	18.12	0.08

**Table 2:** Statistical results of the response options for each WB item.

	Response Options for WB (Subjects/Proportion (%))					
Items for Measuring WB	Always	Often	Sometimes	Seldom	Never/Almost Never	Mean ± SD
Is your work emotionally exhausting?	38 (2.33)	98 (6.00)	549 (33.62)	715 (43.78)	233 (14.27)	34.58 ± 22.11
2. Do you feel burnt out because of your work?	22 (1.35)	79 (4.84)	471 (28.84)	803 (49.17)	258 (15.80)	31.69 ± 20.77
3. Does your work frustrate you?	14 (0.86)	43 (2.63)	543 (33.25)	810 (49.60)	223 (13.66)	31.86 ± 18.98
4. Do you feel worn out at the end of the working day?	68 (4.16)	178 (10.90)	680 (41.64)	568 (34.78)	139 (8.51)	41.86 ± 23.16
5. Are you exhausted in the morning at the thought of another day at work?	61 (3.74)	124 (7.59)	524 (32.09)	718 (43.97)	206 (12.61)	36.47 ± 23.41
6. Do you feel that every working hour is tiring for you?	18 (1.10)	41 (2.51)	349 (21.37)	896 (54.87)	329 (20.15)	27.39 ± 19.46
7. Do you have enough energy for family and friends during leisure time?	261 (15.98)	646 (39.56)	528 (32.33)	165 (10.10)	33 (2.02)	35.66 ± 23.56

Cronbach’s alpha = 0.87; SD, standard deviation.

**Table 3:** Stratified analysis of work-related burnout and alcohol use.

		WB		AUF	
Survey Variables	N	β	*p*	β	*p*
***Work experience***					
WE	1615	−0.23	***	−0.21	**
***Marriage state***					
Married	779	−4.30	***	−2.68	*
Unmarried	836	1.00		1.00	
***Parenthood***					
Yes	703	−4.86	***	−3.10	*
No	912	1.00		1.00	
***Leisure activity with family and friends***					
mean score	1615	−014	***	−0.00	
***Exercise habit weekly***					
REW	933	−5.60	***	1.72	
None REW	682	1.00		1.00	
***SLD (per day) ranks***					
<6 h	626	6.27	***	3.11	*
>6 h	989	1.00		1.00	
***Education degree***					
Master’s degree or above	297	−2.21	*	2.17	
University or below university degree	1318	1.00		1.00	
***Overtime work per month***					
Experience OT	561	8.88	***	4.33	**
Seldom OT	1054	1.00		1.00	
***Shift schedules***					
IRS work	192	8.87	***	3.02	
RS work	196	6.23	***	0.29	
Night shift work	166	3.24	*	3.66	
DS work	1061	1.00		1.00	
***Professional fields***					
Physicians	138	9.89	***	9.96	***
Nurses	613	8.44	***	1.30	
PTs	283	2.17		3.11	
ADs	581	1.00		1.00	
***Suffering CD***					
Yes	638	3.47	***	3.01	*
No	977	1.00		1.00	
***MS pain***					
NBSP	1615	6.30	***	2.48	**
BAP	1615	1.44	**	0.19	
BKP	1615	0.75		−0.44	

N, participants; β, the linear regression coefficient; * *p* < 0.05, ** *p* < 0.01, *** *p* < 0.0001.

**Table 4:** AU effect in the linear regression models of WB.

	M_0_		M_1_		M_2_	
Main Effect	β	*p*	β	*p*	β	*p*
AUF	0.07	***	0.04	**	0.03	

β, the linear regression coefficient; ** *p* < 0.01, *** *p* < 0.0001; M_0_, without adjustment for variables; M_1_, adjustment for work experience, marriage status, parenthood, leisure activity with family and friends, exercise habit weekly, sleep duration, educational level, overtime work per month, shift schedules, professional fields, and CD; M_2_, adjustment for all the variables included in M_1_ and an additional variable, namely, NBSP.

**Table 5:** Mediation effect of AUF on the relationship between NBSP and WB.

		WB						
IV	M	c′	A	s_a_	b	s_b_	Z	MP (%)
NBSP	AUF	6.19 ***	2.46 **	0.67	0.05 **	0.02	2.07 *	1.95
AUF	NBSP	0.05 **	0.00 **	0.00	6.19 ***	0.41	3.25 **	27.08

* *p* < 0.05, ** *p* < 0.01, *** *p* < 0.0001; M, mediation factor; c′, the direct effect of NBSP on WB; a, the first-stage effect of NBSP on AU; s_a_, the standard error for a; b, the second stage effect for AUF on WB; s_b_, the standard error for b; MP, mediation proportion.

## Data Availability

No additional data are available.
